# Supplementary material for: Genetic regulation of liver lipids in a mouse model of insulin resistance and hepatic steatosis
Source: Mol Syst Biol. 2021 Jan 8;17(1):e9684. doi: 10.15252/msb.20209684 (PMC7792507; doi:10.15252/msb.20209684)
Supplement: Supplementary file 1 — Expanded View Figures PDF [file MSB-17-e9684-s001.pdf]

Expanded View Figures

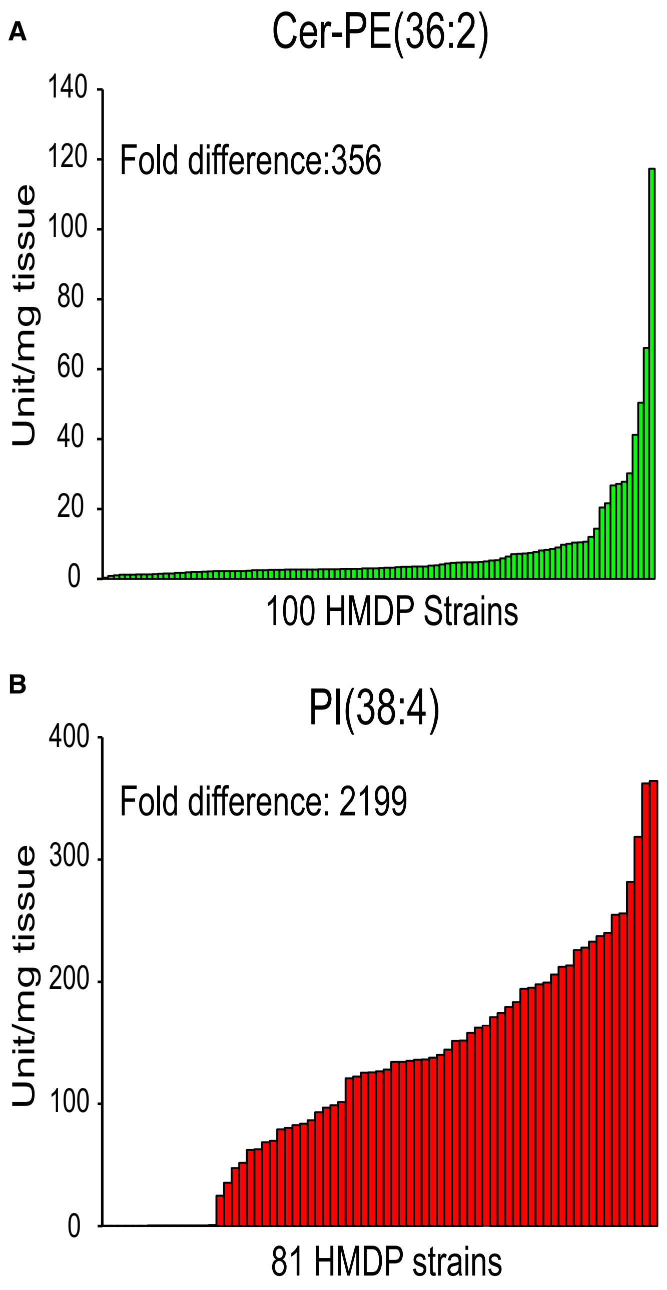

Figure EV1. Highly variable lipids within the HMDP.

A, B Ceramide-phosphatidylethanolamine 36:2 (Cer-PE(36:2)) and phosphatidylinositol 38:4 (PI(38:4)) are examples of hepatic lipid species with a substantial variation among the mice strains across the HMDP. Cer-PE(36:2) showed measured levels in 100 out of 101 HMDP strains. PI (38:4) showed measured levels in 81 out of 101 HMDP strains.

© 2021 The Authors

Figure EV2. WGCNA analysis and correlations with microbiota.

WGCNA was performed to dissect which lipids segregated into modules and correlated with microbiota abundances. Samples were first arranged by hierarchical clustering to detect outliers, where a height of 2,100 (red line) was used as a cutoff for inclusion (top left). Scale-free topology (top middle) and mean connectivity (top right) are also provided for analysis. Microbes were summarized at the levels of order (o), genus (g), or family (f). Heatmap showing the lipid modules (*y*-axis) and correlation with type of microbiome abundances (*x*-axis). $^*P < 0.001$. *P*-values were calculated based on significance of regression (students test) and adjusted for multiple comparisons (FDR = 0.05).

© 2021 The Authors

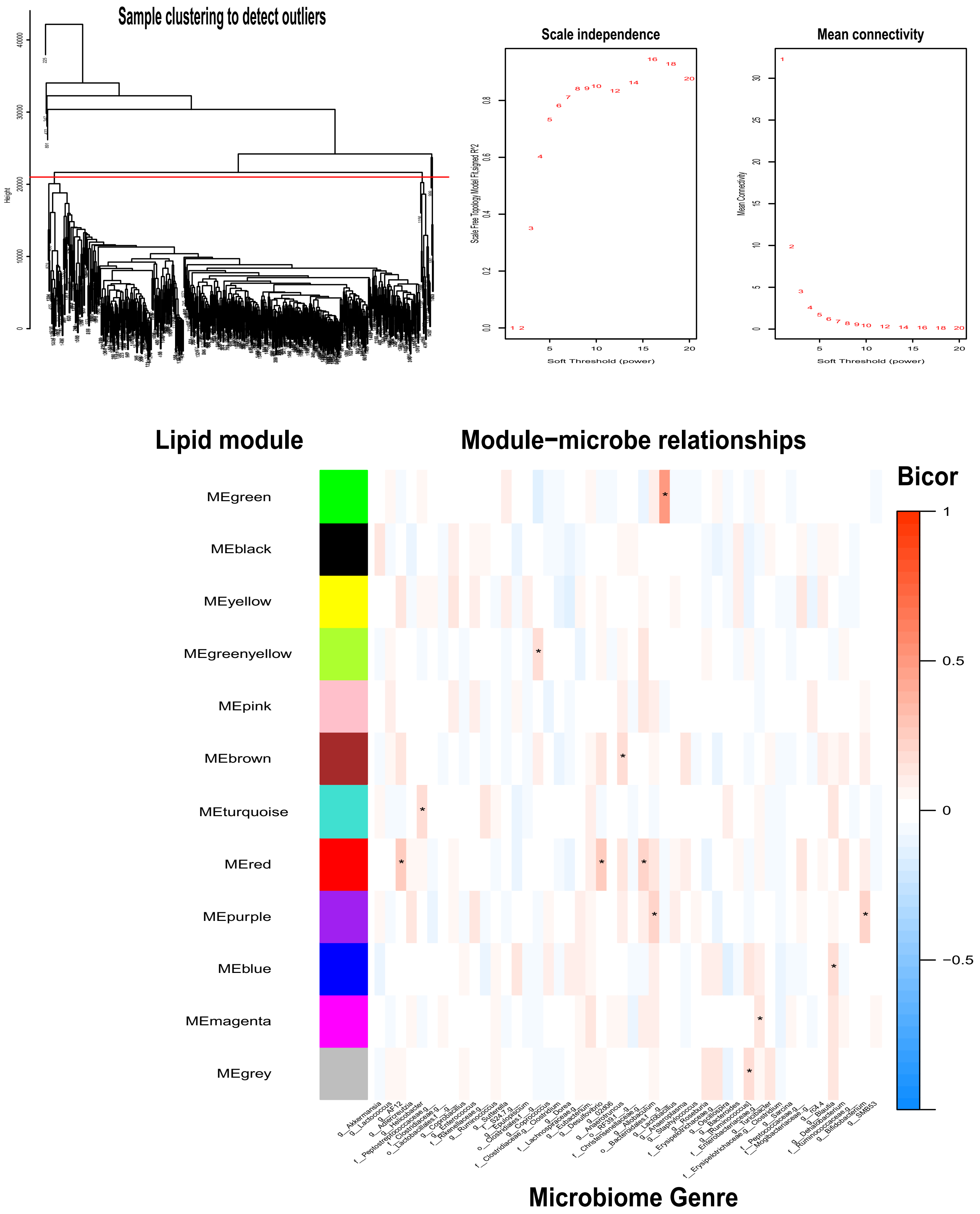
Sample clustering to detect outliers
Height
Scale independence
Scale Free Topology Model Fit,signed R^2
Soft Threshold (power)
Mean connectivity
Mean Connectivity
Soft Threshold (power)
Lipid module
Module−microbe relationships
MEgreen
MEblack
MEyellow
MEgreenyellow
MEpink
MEbrown
MEturquoise
MEred
MEpurple
MEblue
MEmagenta
MEgrey
Bicor
1
0.5
0
−0.5
Microbiome Genre

Figure EV2.

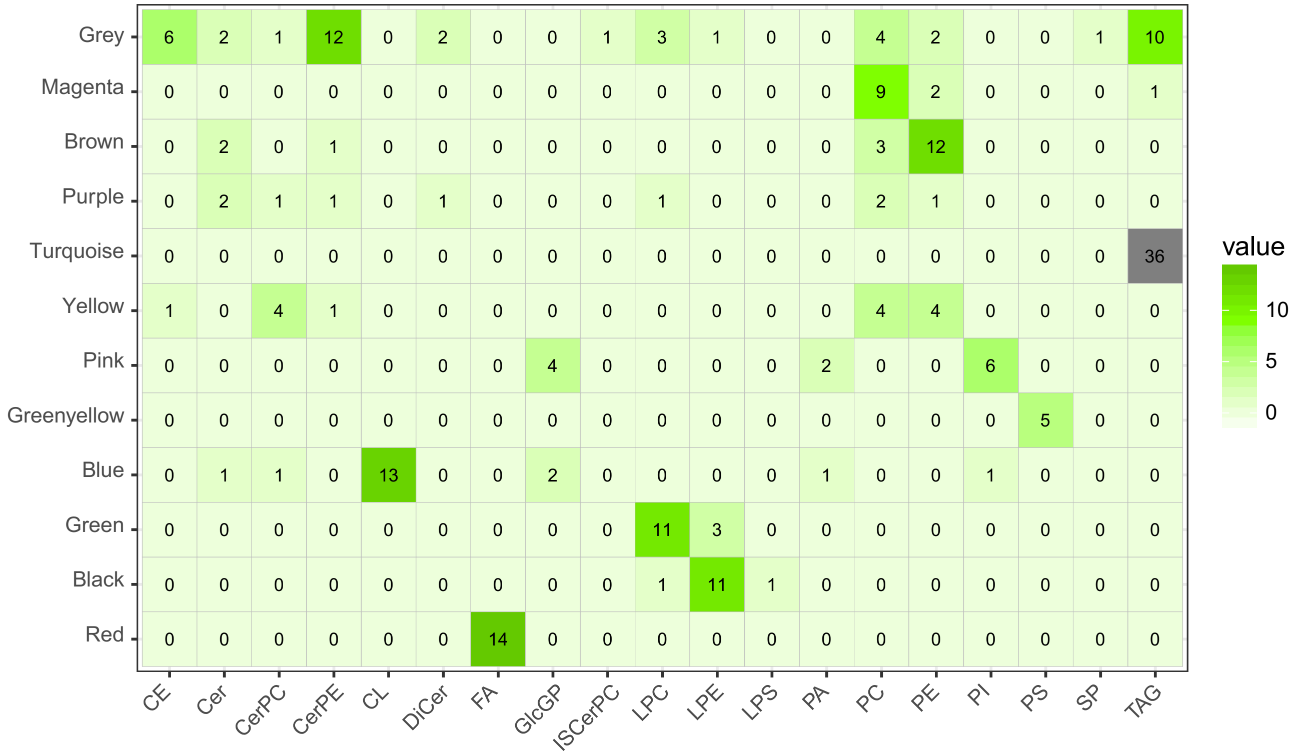

	CE	Cer	CerPC	CerPE	CL	DiCer	FA	GlcGP	ISCerPC	LPC	LPE	LPS	PA	PC	PE	PI	PS	SP	TAG
Grey	6	2	1	12	0	2	0	0	1	3	1	0	0	4	2	0	0	1	10
Magenta	0	0	0	0	0	0	0	0	0	0	0	0	0	9	2	0	0	0	1
Brown	0	2	0	1	0	0	0	0	0	0	0	0	0	3	12	0	0	0	0
Purple	0	2	1	1	0	1	0	0	0	1	0	0	0	2	1	0	0	0	0
Turquoise	0	0	0	0	0	0	0	0	0	0	0	0	0	0	0	0	0	0	36
Yellow	1	0	4	1	0	0	0	0	0	0	0	0	0	4	4	0	0	0	0
Pink	0	0	0	0	0	0	0	4	0	0	0	0	2	0	0	6	0	0	0
Greenyellow	0	0	0	0	0	0	0	0	0	0	0	0	0	0	0	0	5	0	0
Blue	0	1	1	0	13	0	0	2	0	0	0	0	1	0	0	1	0	0	0
Green	0	0	0	0	0	0	0	0	0	11	3	0	0	0	0	0	0	0	0
Black	0	0	0	0	0	0	0	0	0	1	11	1	0	0	0	0	0	0	0
Red	0	0	0	0	0	0	14	0	0	0	0	0	0	0	0	0	0	0	0

Figure EV3. Module membership by lipid class. Heatmap showing the number of lipids within a class per module.

WGCNA was performed to dissect which lipids segregated into modules and correlated with traits (body fat %, body weight, HDL, LDL, liver total cholesterol, liver mitochondria, liver total phosphatidyl cholesterol, plasma free fatty acids, plasma glucose, plasma insulin, and plasma triacylglycerol). The darker the color, the more lipids per module, with the highest membership shown in gray. CE, cholesterol esters; Cer, ceramides; CerPC, ceramide phosphatidylcholines; Cer-PE, ceramide-phosphatidylethanolamines; CL, cardiolipins; DiCer, dihydroceramides; FA, free fatty acids; GlcGP, glycosylglycerophospholipids; ISCerPC; IS-ceramide phosphatidylcholines; LPC, lysophosphatidylcholines; LPE, lysophosphatidylethanolamines; LPS, lysophosphatidylserines; PA, phosphatidic acid; PC, phosphatidylcholines; PE, phosphatidylethanolamines; PI, phosphatidylinositols; PS, phosphatidylserines; SP, sphingolipids; TAG, triacylglycerols.

Figure EV4. Undirected network of interactions between hepatic lipids and indicated phenotypic traits in 101 strains of mice fed a HF/HS diet.

Nodes show either individual lipid species or phenotypic traits, where color indicators are given in the figure. Edges are connected between nodes with a significant correlation ($P < 0.01$), with the distance reflecting increasing significance of correlation. *P*-values were calculated based on significance of regression (Student's test) and adjusted for multiple comparisons (FDR = 0.05) CE, cholesterol esters; Cer, ceramides; CerPC, ceramide phosphatidylcholines; Cer-PE, ceramide-phosphatidylethanolamines; CL, cardiolipins; DiCer, dihydroceramides; FA, free fatty acids; GlcGP, glycosylglycerophospholipids; LPC, lysophosphatidylcholines; LPE, lysophosphatidylethanolamines; LPS, lysophosphatidylserines; PA, phosphatidic acid; PC, phosphatidylcholines; PE, phosphatidylethanolamines; PI, phosphatidylinositols; PS, phosphatidylserines; TAG, triacylglycerols.

© 2021 The Authors

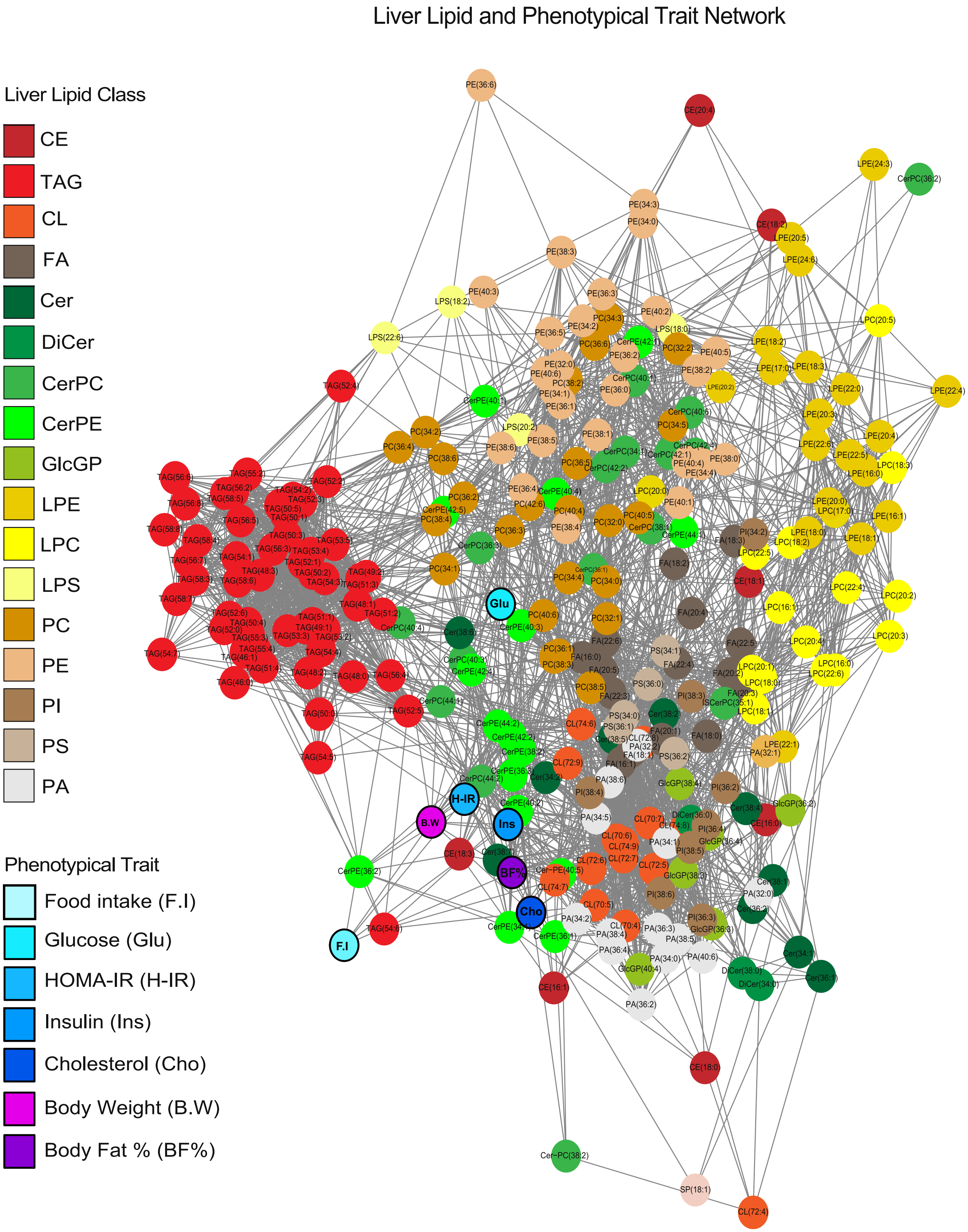
Liver Lipid and Phenotypical Trait Network
Liver Lipid Class
CE
TAG
CL
FA
Cer
DiCer
CerPC
CerPE
GlcGP
LPE
LPC
LPS
PC
PE
PI
PS
PA
Phenotypical Trait
Food intake (F.I)
Glucose (Glu)
HOMA-IR (H-IR)
Insulin (Ins)
Cholesterol (Cho)
Body Weight (B.W)
Body Fat % (BF%)

Figure EV4.

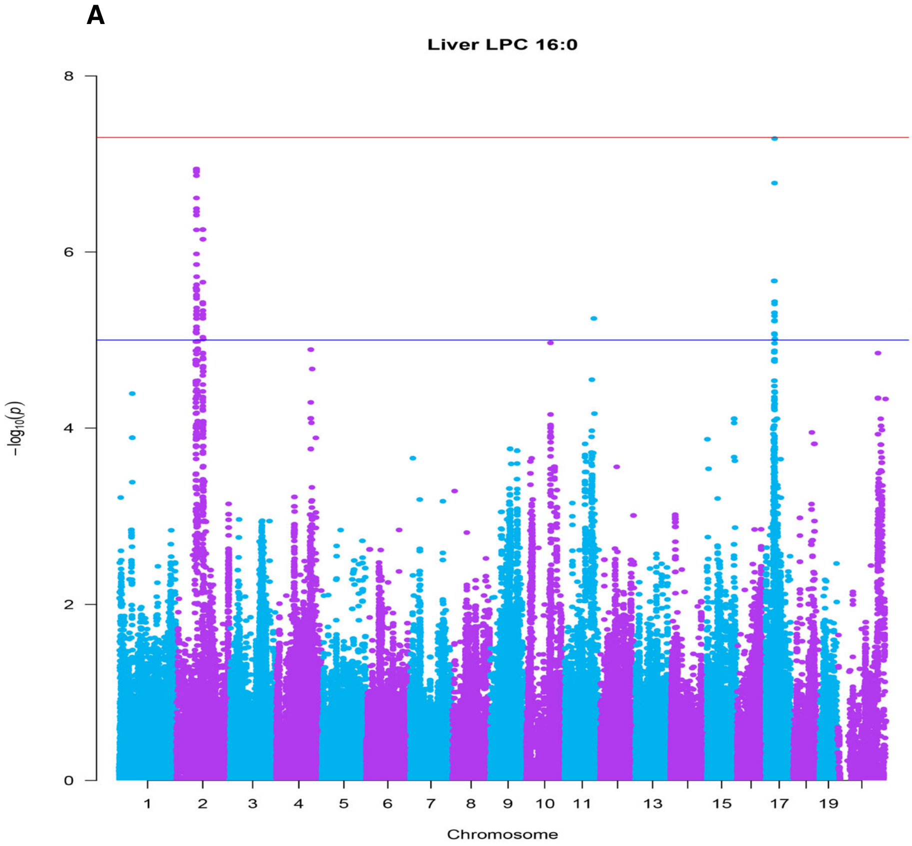

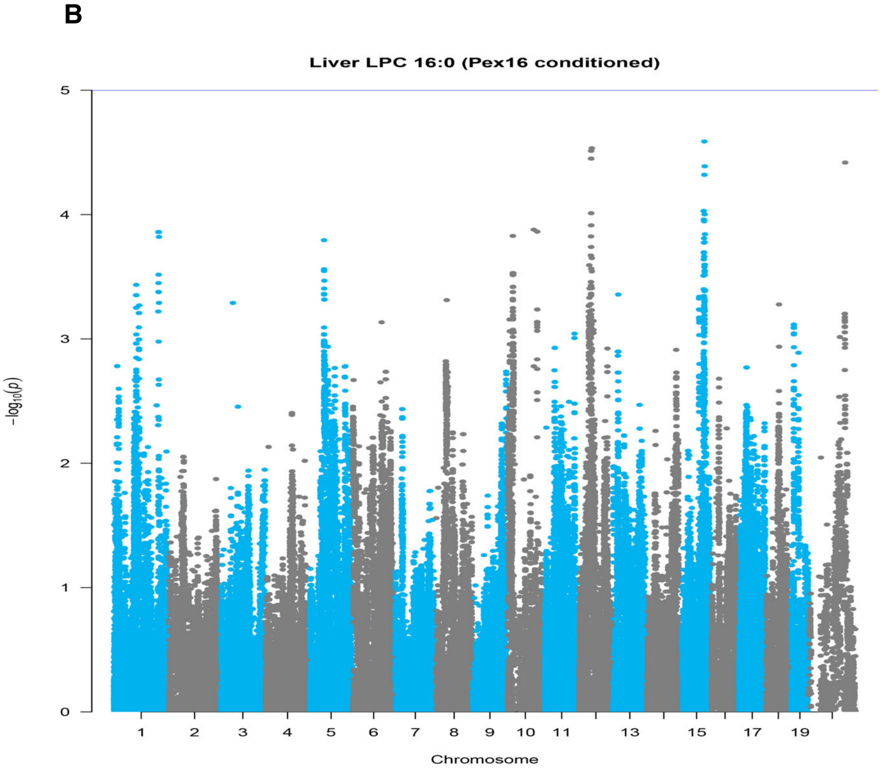

Figure EV5. Mediation example.

A GWAS for levels of hepatic LPC (16:0), where the locus on Chr2 comapped to a cis-eQTL for Pex16. Red line shows Bonferroni-corrected threshold, and blue shows an FDR = 0.01 *P*-value of significance calculated based on FaST-LMM *P*-values.

B The same GWAS as in (A), with the exception of adding expression levels of Pex16 as a covariate to the linear mixed model. The locus on Chr2 was significantly lower in its association *P*-value.

 © 2021 The Authors

A Genome Browser View

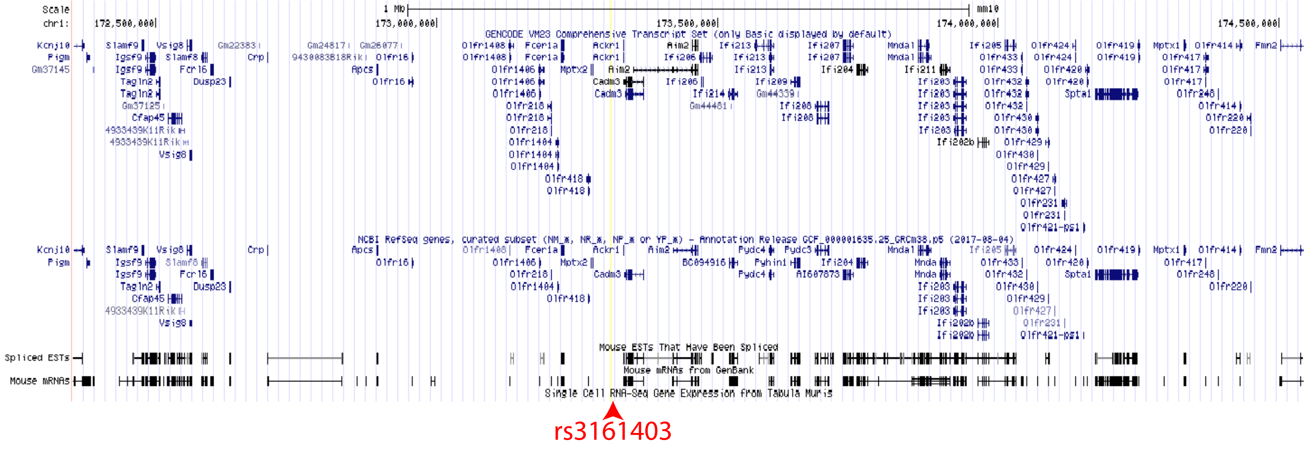

B Association between rs3161403 and genes detected in arrays

probesetID	gene_symbol	pvalue	snp_weight
1448775_at	Ifi203	3.11E-22	-1.22051
1450063_at	Fmn2	4.27E-10	0.756813
1450064_at	Fmn2	2.08E-09	0.459566
1419059_at	Apcs	3.82E-09	0.404058
1419603_at	Ifi204	2.05E-07	-0.105633
1421946_at	Crp	3.73E-07	-0.0906825
1425120_x_at	Ifi27l2b	0.01515	0.183407
1418921_at	Cadm3	0.02577	0.0314399
1424254_at	Ifitm1	0.0446	0.0651126
1425294_at	Slamf8	0.06145	-0.0299238
1450301_at	Ifi44l	0.0802	-0.0147574
1440865_at	Ifitm6	0.11549	0.0319731
1424617_at	Ifi35	0.15716	-0.0365164
1422476_at	Ifi30	0.17034	0.0326649
1417292_at	Ifi47	0.18131	-0.0671039
1450783_at	Ifit1	0.21635	-0.10185
1417735_at	Mptx1	0.38884	0.00942848
1421277_at	Spta1	0.42335	0.0100043
1422112_at	Ackr2	0.43614	0.0119503
1449272_at	Cadm3	0.60012	-0.00741335
1422111_at	Ackr2	0.6467	-0.00521301
1426278_at	Ifi27l2a	0.6697	-0.0334925
1423754_at	Ifitm3	0.70354	-0.0119484
1418293_at	Ifit2	0.73458	0.0125386
1421596_s_at	Ifi44l	0.82664	0.0121554
1423555_a_at	Ifi44	0.87333	0.01157
1426276_at	Ifih1	0.95079	-0.00230309
1421278_s_at	Spta1	0.98283	0.0002817

Figure EV6. Ifi203 Locus data.

A Genome browser view of locus containing the peak SNP (rs3161403) for hepatic PC(C38:3) and surrounding genes.

B Fast-LMM cis-eQTL *P*-value and SNP weight for each gene observed in (A) and detected in liver expression arrays. The list is ordered by increasing *P*-value of association. *P*-values of significance calculated based on FaST-LMM *P*-values.

© 2021 The Authors
